# Supplementary material for: “A sense of the bigger picture:” A qualitative analysis of follow-up interviews with people with bipolar disorder who self-reported psilocybin use
Source: PLoS One. 2022 Dec 14;17(12):e0279073. doi: 10.1371/journal.pone.0279073 (PMC9749989; doi:10.1371/journal.pone.0279073)
Supplement: S1 File — (PDF) [file pone.0279073.s001.pdf]

S1 File. Qualitative Interview Guide.

Why do people with bipolar disorder use psilocybin?

1. How often have you used magic mushrooms?
    a. *If once:* Did something happen that first time that made you not want to use them again?
2. Researchers don't know very much about why people with bipolar disorder choose to use magic mushrooms. I'm wondering, why have you used magic mushrooms?
    a. What have your goals and intentions been for using magic mushrooms?
        i. *[Follow-up with specific intentions endorsed]*
        ii. Have you ever used mushrooms to manage your bipolar symptoms?
            1. *If yes:* How has it compared to other medications you have tried? To other mental health treatments you've tried?

What are the circumstances in which people with bipolar disorder use psilocybin?

3. In what settings or situations have you used psilocybin?
    a. *Follow-up:* Have you used alone? Have you used socially? Have you used with a professional guide/therapist? At a party, festival, or social gathering?
    b. *Follow-up:* What mental or emotional states have you been in when using psilocybin (e.g., Have you used when you've been depressed, when you've felt emotionally level, when you've felt hypomanic or manic...?)
    c. *Follow-up:* Have you ever mixed mushroom use with other drugs? What was that experience like for you?

4. Researchers know very little about how people manage their psychiatric medications while using mushrooms in the real world. Have you ever used mushrooms during a time when you were being prescribed psychiatric medications?
    a. *If yes:* What medications were you prescribed? What happened when you used mushrooms?
    b. *If yes:* Did you make adjustments to how you took your psychiatric meds either before, during, or after using mushrooms? If so, which meds? What led you to do that?

What are the themes surrounding what occurs when psilocybin is used?

5. Researchers are still unsure whether psilocybin use leads to any changes in the type, frequency, or intensity of bipolar symptoms. Have there been any shifts in your symptoms during or after magic mushroom use?
    a. *If yes:* What is your experience of (hypo)mania? What happens to your symptoms when using psilocybin?
    b. *Follow-up:* Has it made them better or worse? Can you provide examples? What do you think accounts for the *[increase/decrease]*?
    c. *If appropriate:* What has happened to your depressive symptoms when you have used psilocybin?
        i. *Follow-up:* Has it made them better or worse? Can you provide examples? What do you think accounts for the *[increase/decrease]*?
    d. *If appropriate:* What has happened regarding psychotic symptoms when you have used psilocybin?

        i. *Follow-up:* Did you experience any psychotic symptoms in the day(s) following psilocybin use?
            1. *If yes:* What happened? How have you managed them?

6. *Review survey endorsements of sleep issues:*
    a. *If yes:* I saw on the survey you noted some sleep related issues in the weeks following mushroom use. What happened? Do you suppose the sleep issues you experienced related to the mushroom trip? How so? What happened with your mood and mental health during that time?
    b. *If no:* I saw on the survey you denied having issues falling or staying asleep in the weeks following mushroom use. What is your sleep typically like after using mushrooms? Has anything abnormal occurred for you?
7. *Review survey endorsements of ED visit, psychiatric, or medical hospitalizations:*
    a. *If yes:* I saw on the survey you noted experience with *[ED, hospital]* in the weeks following mushroom use. What happened that led to that? Do you suppose the visit/hospitalization was related to your mushroom experience? What makes you say [*yes/no*]?
    b. *If no:* Have you ever visited the ED or hospital within a couple weeks following mushroom use?
    c. *If yes:* What happened? Do you think this was related to the mushroom trip?
8. Has using psilocybin made you think differently about your bipolar disorder?
    a. *Follow-up:* Have mushrooms changed your relationship with your symptoms?

What are the intentions for future psilocybin use among people with bipolar disorder?

9. If you could give advice about using magic mushrooms to someone else with your same mental health picture, what would you tell them?
