# Supplementary material for: “A sense of the bigger picture:” A qualitative analysis of follow-up interviews with people with bipolar disorder who self-reported psilocybin use
Source: PLoS One. 2022 Dec 14;17(12):e0279073. doi: 10.1371/journal.pone.0279073 (PMC9749989; doi:10.1371/journal.pone.0279073)
Supplement: Attachment — Submitted filename: Response to Reviewers.pdf [file pone.0279073.s002.pdf]

**MEGHAN DELLACROSSE**

mdellacrosse@wi.edu | 646-853-1760

Sept 28, 2022

Dr. Sidarta Ribeiro
Academic Editor
PLOS ONE

Dear Dr. Sidarta Ribeiro:

Thank you for your response, and for providing the opportunity to submit a revised draft of our manuscript *"A sense of the bigger picture:" A qualitative analysis of follow-up interviews with people with bipolar disorder who self-reported psilocybin use* to PLOS ONE. We value the time that you and the reviewers have contributed to providing feedback on our manuscript, which we found to be equally thoughtful as thorough. We are grateful for the insightful comments provided and have been able to incorporate changes that reflect the majority of suggestions provided by the reviewers. I have highlighted the changes in the manuscript using track changes, and also included an unmarked copy of that document, as requested.

In addition to the items you specified in your email regarding style requirements, our ethics, competing interests, and data availability statements, please see below for a point-by-point response to the reviewer's comments and feedback. Note, text in bold and italics indicates the original requests in the body of your email, as well as reviewer's comments and feedback.

1. ***Please ensure that your manuscript meetings PLOS ONE's style requirements.***
    a. Response: I have reviewed PLOS ONE's style requirements, including file naming, and confirm our manuscript and corresponding materials conform to the templates provided.
2. ***Please amend your current ethics statement to address the following concerns: a) Did participants provide their written or verbal informed consent to participate in this study? b) If consent was verbal, please explain i) why written consent was not obtained, ii) how you documented participant consent, and iii) whether the ethics committees/IRB approved this consent procedure.***
    a. Response: Written informed consent was collected for all participants in Phase II qualitative interview. The consent was approved by UCSF IRB (IRB#20-31330).
3. ***Thank you for stating the following in the Competing Interests section:***
   ***"EEM has received support for patient educational events from Otsuka-Lundbeck Foundation. JW is currently consulting on scientific protocol development for Alvarius Pharmaceuticals, and previously consulted on protocol development for Psilo Scientific Ltd. and Silo Pharma. This does not in any way alter our adherence to PLOS publishing policies."***
   ***Please confirm that this does not alter your adherence to all PLOS ONE policies on sharing data and materials, by including the following statement: "This does not alter our adherence to PLOS ONE policies on sharing data and materials." If there are restrictions on sharing of data and/or materials,***

***please state these. Please note that we cannot proceed with consideration of your article until this information has been declared. Please include your updated Competing Interests statement in your cover letter; we will change the online submission form on your behalf.***

a. Response: These disclosures do not in any way alter our adherence to PLOS publishing policies on sharing data and materials. Complete transcript data of qualitative interviews analyzed in this paper cannot be shared publicly due to clear ethical reasons. First, the interviewees have not given consent for this. Additionally, although we did remove personal identifiers from the interview transcripts (e.g., names, locations), given the unique narratives disclosed by individuals with a potentially stigmatized diagnosis about use of a controlled substance, the raw interview transcripts may contain sensitive and identifiable information. Therefore, participant confidentiality must be protected. We can provide access to the transcripts subject to certain conditions. Requests can be made via email to the 1) corresponding author, mdellacrosse@wi.edu, or 2) the Translational Psychedelic Research Program (TrPR) at UCSF, psilocybinstudies@ucsf.edu.

4. ***In your Data Availability statement, you have not specified where the minimal data set underlying the results described in your manuscript can be found. PLOS defines a study's minimal data set as the underlying data used to reach the conclusions drawn in the manuscript and any additional data required to replicate the reported study findings in their entirety. All PLOS journals require that the minimal data set be made fully available. For more information about our data policy, please see*** http://journals.plos.org/plosone/s/data-availability.

***Upon re-submitting your revised manuscript, please upload your study's minimal underlying data set as either Supporting Information files or to a stable, public repository and include the relevant URL's, DOIs, or accession numbers within your revised cover letter. For a list of acceptable repositories, please see*** http://journals.plos.org/plosone/s/data-availability#loc-recommended-repositories.

***Important: If there are ethical or legal restrictions to sharing data publicly, please explain these restrictions in detail. Please see our guidelines for more information on what we consider unacceptable restrictions to publicly sharing data:*** http://journals.plos.org/plosone/s/data-availability#loc-unacceptable-data-access-restrictions. ***Note that it is not acceptable for the authors to be the sole named individuals responsible for ensuring data access. We will update your Data Availability statement to reflect the information you provide in your cover letter.***

a. Response: There are ethical restrictions to sharing raw transcript data. As described in above items 3.a., a detailed explanation of these restrictions is as follows: Complete transcript data of qualitative interviews analyzed in this paper cannot be shared publicly due to clear ethical reasons. First, the interviewees have not given consent for this. Additionally, although we did remove personal identifiers from the interview transcripts (e.g., names, locations), given the unique narratives disclosed by individuals with a potentially stigmatized diagnosis about use of a controlled substance, the raw interview transcripts may contain sensitive and identifiable information. Therefore, beyond the quoted in-text transcript content, we are only able to provide access to the transcripts subject to certain conditions. Requests can be made via email to the 1) corresponding author, mdellacrosse@wi.edu, or 2) the Translational Psychedelic Research Program at UCSF, psilocybinstudies@ucsf.edu.
b. It is our understanding that the combination of these ethical limitations along with excerpted in-text material meets the PLOS data sharing policy as described in the Guidelines for qualitative data.

5. ***Your abstract cannot contain citations. Please only include citations in the body text of the manuscripts, and ensure that they remain in ascending numerical order on first mention.***
    a. Response: Yes, agreed. This confirms there are no citations contained in the abstract.

Comments from Reviewer 1

INTRODUCTION

***Page 3, lines 56-57. What do you mean by 'low efficacy'? Please be a bit more precise about the issue.***

- Response: Thank you for this clarifying question. "Low efficacy" has been modified to more apt "inconsistent efficacy" as reflected in (Gitlin, 2018).
    - Reference:
    - Gitlin MJ. Antidepressants in bipolar depression: an enduring controversy. International journal of bipolar disorders. 2018;6(1):1-7.

***Pg. 4, ln. 66. To the best of my knowledge, ritual use of psilocybin-containing mushrooms has only been documented with certainty among the Mazatecs of Mexico. Please double-check that the referenced text is neither unduly generic nor 'generous' with ethnobotanical data.***

- Response: Thank you for your remark. We have added a second complementary reference that also discusses historical context for psilocybin-containing mushrooms.

***Pg. 5, ln. 87. It is unclear at this point why the evidence that psilocybin actually induces TEAS is not 'convincing'. Only in the next paragraph is this made clear. Please modify this sentence to fit inside the text's argumentative thread.***

- Response: Thank you for this helpful feedback. This sentence has been adjusted to more clearly frame the problem and anticipate the following paragraph.

***Pg. 9, ln. 193. The text reads 'Audio-recorded Phase II', but previously (pg. 9, ln. 176) it is stated that interviews were 'audio-video recorded'. Please clarify.***

- Response: Thank you for catching this typo; both recordings were audio-video. The text has been adjusted accordingly.

RESULTS

***Pg. 11, Table 1. Since participant 114 identify themself as gender-queer, why not refer to them as 'Latinx' rather than 'Latino'?***

- Response: Thank you for this keen observation. The racial/ethnic identity category was a self-described write-in category. Therefore, "Latino" was how this participant self-identified. Your observation highlighted that the "write-in" specifier on the table may not have been entirely clear, thus we have modified this as "self-described" to provide clarity for the reader. Salinas (2020) highlights the need for cultural humility with regard to identity labels (e.g., Latinx) given that experiences and identifications vary despite intentions of inclusivity. Our choice to provide self-report options for both gender and race/ethnicity categories was determined by consultation with various community groups.
    - Reference:
    - Salinas Jr C. The complexity of the "x" in Latinx: How Latinx/a/o students relate to, identify with, and understand the term Latinx. Journal of Hispanic Higher Education. 2020;19(2):149-68.

***Pg. 14, p. 277. Please briefly define here or in the discussion (pg. 28, p 603) what is emotion processing.***

- Response: - Thank you for this comment; as requested, a brief definition has been provided on page 14 in addition to context on page 28.

***Pg. 16, lns. 319-22. The definition of integration - a commonly used but vaguely defined term in the field of psychedelic research - is unclear, as is the type of integration process that occurred in the case of participant 112.***

- Response: Thank you for your helpful observation and comment. We have removed the phrase "integration phase" from page 16 of the manuscript text as the "integration process" that the participant is referring to is actually specific to the timeframe following psilocybin use in naturalistic settings. We agree that the word "integration" is often vaguely used in psychedelic research, most commonly in relation to psychedelic-assisted psychotherapy (PAP) (Earleywine et al., 2022). In this instance, however, the participant is independently referring to the days and weeks following the psilocybin drug experience and not a formal process of integration more strongly associated with PAP and/or clinical trials for these drugs. The interview guide used in this study did not inquire about integration, however, we hope that future research will investigate this.
  - References:
  - Earleywine M, Low F, Lau C, De Leo J. Integration in Psychedelic-Assisted Treatments: Recurring Themes in Current Providers' Definitions, Challenges, and Concerns. Journal of Humanistic Psychology. 2022 Apr 2:00221678221085800.

***Pg. 19, lns. 393-4. Participant 115 is described in Table 1 as BD1, but in this passage she describes hallucinations, a psychotic feature. Please clarify if these symptoms were self-limiting and/or why she was not diagnosed with BD with psychotic characteristics.***

- Response: Thank you for your clarifying question. The "disturbing auditory hallucinations" described in the section you are referring to was limited to this individual's drug experience of psilocybin and therefore would not have been captured by the diagnostic criteria to indicate BD with psychotic features.

OTHER FEEDBACK, REVIEWER 1:

***Responses of persons with BD to mushrooms containing psilocybin vary significantly. I missed a table that indicated how each participant was included or excluded from each category/theme. I understand that this type of resource would assist us in better understanding what relationships may exist, for example, between having a challenging experience or not and presenting mood improvement or sleep effects (whether insomnia or improved sleep) afterward - and whether such relationships exist. Please include this table or explain why it should not be included.***

- Response: Thank you for your feedback and sharing how you feel that a table would have assisted your experience. We agree that tables can assist in presenting categories and themes in certain research contexts, as well as make accessible qualitative findings. Cloutier and Ravasi (2021) highlight the ways tables can organize, analyze, and display qualitative research findings. However, given that participants in our study have had multiple experiences they have referred to, and both good/bad experiences, we have opted not to make a table as we believe it would cause confusion. Furthermore, this decision was also based on a belief that such a table may encourage the reader to look for associations between demographic details and experiences, which is not what we set out do with this small qualitative sample. One of our aims in this exploration here was to utilize thematic analysis to identify and describe subjective experiences and generate initial hypotheses in an understudied population. Certainly, it is our hope that future studies in psychedelic research will be designed to further understanding of relationships across positive/challenging experiences, mood changes, and sleep.
  - Reference:
  - Cloutier C, Ravasi D. Using tables to enhance trustworthiness in qualitative research. Strategic Organization. 2021;19(1):113-33.

***The 'Future directions' section (pg. 33) appears to be a little premature in offering solutions to the problem of BD interaction with psilocybin consumption, especially for qualitative research that, as the text itself states, should raise questions rather than provide answers. Before outlining how psilocybin may be given to persons with BD in clinical trials, I believe you should specify what types of study could and should be conducted - not to mention the ethical aspects - to address this issue.***

- Response: Thank you for these thoughtful remarks, which were used to inform several modifications to this section, including specifying a small feasibility trial designed to evaluate safety and useability in a controlled setting. Additionally, we have specified the age range of 30-65 that we believe would help to minimize safety risks. for participants in a safety trial, which is informed by onset age for individuals with bipolar disorder (Bolton et al., 2022) to minimize risk. (See lines 801-806.)
    - Reference:
    - Bolton S, Warner J, Harriss E, Geddes J, Saunders KE. Bipolar disorder: Trimodal age-at-onset distribution. Bipolar disorders. 2021;23(4):341-56.

***Finally, data availability was not mentioned in the text, as far as I could tell. Please specify where you plan to publish the interview transcripts or provide a justification—such as ethical concerns—for why you are unable to do so.***

- Response: Thank you for requesting specification of ethical concerns for sharing full transcripts. To address this issue, our statement about data availability has been expanded on as follows:
    - "Complete transcript data of qualitative interviews analyzed in this paper cannot be shared publicly for clear ethical reasons. First, the interviewees have not given consent for this. Additionally, although we did remove personal identifiers from the interview transcripts (e.g., names locations), given the unique narratives disclosed by individuals with a potentially stigmatized diagnosis about use of a controlled substance, the raw interview transcripts may contain sensitive and identifiable information. Therefore, participant confidentiality must be protected."

Comments from Reviewer 2

***This study consists of qualitative analysis of interviews with bipolar disorder (BD) patients who engaged in the use of psychedelics. This article has several limitations, and most of them are acknowledged by the authors. I will not criticize the limitations intrinsic to the method, but try to raise concerns that can improve the quality of the manuscript. I leave to the Editor the decision of whether purely qualitative analyses are within the scope of this journal.***

***Throughout the manuscript, I feel that the authors are somewhat ambiguous about the potential risk of psychedelics in BD patients, which in my opinion is odd given the available evidence. Thus, in the introduction they state that "little evidence" is usually given to exclude BD patients from trials. In the next paragraphs the authors question the exclusion of BD patients based on the possibility of TEAS. Yet in the next paragraph a study of a small sample of subjects suggests that BD patients are indeed at risk of developing manic episodes due to psilocybin. Perhaps most surprising is the study by Morton et al. cited below, which states that 541 participants who self-reported BD, 32% (174) reported worsening of their symptoms, including feelings of mania, anxiety and difficulty sleeping. But near the end of the manuscript, the authors state that the present study demonstrated "clear risks" for this population, yet there is a trial underway to test the safety of psilocybin on BD patients.***

- Response: Thank you for sharing your feedback on the ways you felt that we were somewhat ambiguous about the potential risks of psychedelic use among individuals living with BD. After careful consideration of your comments, we believe that literature review and rationale for our recommendations aligns with available literature and our study findings. We are not sure which line you are referring to

regarding “*little evidence,”* however, on line 82 we stated “little justification” for exclusion is given in the context of psychedelic research.

  - References:
  - Nutt D. Psychedelic drugs—a new era in psychiatry? Dialogues in clinical neuroscience. 2019;21(2):139.
  - Studerus E, Kometer M, Hasler F, Vollenweider FX. Acute, subacute and long-term subjective effects of psilocybin in healthy humans: a pooled analysis of experimental studies. Journal of psychopharmacology. 2011;25(11):1434-52.

- In addition to citing those aforementioned texts that highlight exclusion practices in psychedelic research that lack explanation, we cite literature pertaining to treatment emergent affective switching (TEAS) in the context of treatment for BD depression to underscore unresolved questions in that context. For example, the extent to which the non-static nature of depression in this population challenges study design and contributes to limited treatment options. Some researchers question the extent to which mania following depression is due, perhaps, to the cycling nature of BD itself.
  - References:
  - Jelen LA, Young AH. The treatment of bipolar depression: current status and future perspectives. Current Behavioral Neuroscience Reports. 2020;7(1):1-14.
  - Salvadore G, Quiroz JA, Machado-Vieira R, Henter ID, Manji HK, Zarate Jr CA. The neurobiology of the switch process in bipolar disorder: a review. The Journal of clinical psychiatry. 2010;71(11):1488-501.
  - Licht R, Gijsman H, Nolen W, Angst J. Are antidepressants safe in the treatment of bipolar depression? A critical evaluation of their potential risk to induce switch into mania or cycle acceleration. Acta Psychiatrica Scandinavica. 2008;118(5):337-46.
  - Goldberg JF. How Much Is the Debate Over Antidepressant Efficacy and Safety in Bipolar Depression an Artifact of Study Methodologies? The Journal of Clinical Psychiatry. 2021;82(1):0.
- It is our understanding that your reference to “*study of a small sample of subjects suggests that BD patients are indeed at risk of developing manic episodes due to psilocybin”* was the review of published case reports by Gard and colleagues (2021), referenced beginning on line 97. Those authors identified within a small sample of available case studies salient contextual factors (e.g., poly-substance-use, repeated consumption, and large doses) across cases of serotonergic psychedelic use (e.g., DMT, ayahuasca, LSD, and psilocybin). Out of 17 cases, two described people with history of BD who experienced mania following psychedelic use (note: not psilocybin). It is our belief that, given widespread use (Yockey & King, 2021), and limited treatment options for this population (Jelen & Young, 2020), more research is needed.
  - References:
  - Gard DE, Pleet MM, Bradley ER, Penn A, Gallenstein ML, Riley LS, et al. Evaluating the Risk of Psilocybin for the Treatment of Bipolar Depression: A Review of the Research Literature and Published Case Studies. Journal of Affective Disorders Reports. 2021:100240.
  - Yockey A, King K. Use of psilocybin (“mushrooms”) among US adults: 2015–2018. Journal of Psychedelic Studies. 2021;5(1):17-21.
  - Jelen LA, Young AH. The treatment of bipolar depression: current status and future perspectives. Current Behavioral Neuroscience Reports. 2020;7(1):1-14.
- We believe it may be helpful to highlight (see p. 6) that, although Morton and colleagues found that 32.2% of participants in phase I reported new or emergent symptoms, use of emergency medical services was rare (n=18, 3.3%). Additionally, participants typically reported psilocybin experiences as more helpful than harmful, even in the impacted group.
  - References:
  - Morton E, Sakai K, Ashtari A, Pleet MM, Michalak EE, Woolley J. Risks and benefits of psilocybin use in people with bipolar disorder: An international web-based survey on experiences of ‘magic mushroom’ consumption. (In review). 2022.

***Overall, based on this evidence, my feeling is that 1) there is evidence of adverse effects beyond the anecdotal (Morton et al., 30% adverse effects seems highly significant), 2) there is a plausible candidate mechanism by which these adverse effects manifest (TEAS), 3) there is no discussion concerning how to alleviate these potential risks, or to select a sample with mitigated risk to develop adverse symptoms. In this case, I simply wonder how the risks to the patients will be justified in the upcoming trial.***

- Response: Thank you for your feedback on the overall paper. We appreciate your observations, and that you have referenced findings from Phase I of this study (Morton et al., 2022 [in final review]). Given that the Morton and colleagues (2022) paper is awaiting final publication, we thought it may be helpful to elaborate on some of the findings there. Overall, 32.2% (*n*=174) of respondents (*n*=541) in Phase I reported new or increasing symptoms. The breakdown of these individual symptoms was as follows: 14.2% mania; 10.4% sleep challenges; 9.4% anxiety; 8.9% depression. Of 541 survey participants, 3.3% reported that emergent symptoms necessitated emergency services. As stated in our manuscript on line 129, Morton and colleagues (2022) also highlight that on average survey participants indicated their psilocybin experiences were more helpful than harmful. One limitation of Phase I is that, given aims to understand risk factors for individuals with BD, survey questions did not elicit responses on positive outcomes such as positive symptom changes observed in the qualitative responses in Phase II.
- While we agree that there are risks for this population, it is also indicated that there are potential benefits for people with BD as described in this manuscript's results, as well as in the forthcoming Phase I survey results (Morton et al., 2022). Given limited treatment options for individuals living with this diagnosis (Jelen & Young, 2020), and burden of bipolar depression (Kupka et al., 2007; Sachs et al., 2007), we believe more research is needed. Finally, it is important to note that psilocybin use in naturalistic settings presents greater risk due to factors such as environmental stimuli and polysubstance use, as well as the absence of support and screening. In the context of a controlled clinical trial, such known risk factors can be minimized.
    - References:

    - Morton E, Sakai K, Ashtari A, Pleet MM, Michalak EE, Woolley J. Risks and benefits of psilocybin use in people with bipolar disorder: An international web-based survey on experiences of 'magic mushroom' consumption. (In review). 2022.
    - Jelen LA, Young AH. The treatment of bipolar depression: current status and future perspectives. Current Behavioral Neuroscience Reports. 2020;7(1):1-14.
    - Kupka RW, Altshuler LL, Nolen WA, Suppes T, Luckenbaugh DA, Leverich GS, et al. Three times more days depressed than manic or hypomanic in both bipolar I and bipolar II disorder 1. Bipolar disorders. 2007;9(5):531-5.
    - Sachs GS, Nierenberg AA, Calabrese JR, Marangell LB, Wisniewski SR, Gyulai L, et al. Effectiveness of adjunctive antidepressant treatment for bipolar depression. New England Journal of Medicine. 2007;356(17):1711-22.


***I don't think the criteria to select the subsample was clearly established. Ensuring diversity and representation of minorities might be not enough to select the sample. At the moment, I feel that the process could have been ambiguous, which is not good practice in a study that dramatically reduces the sample size by selecting a subgroup one order of magnitude less than the original.***

- Response: Thank you for your remarks about criteria used for the subsample contained in Phase II interviews, and for specifying the ambiguity you perceived. Within the scope of this study, our decision to use purposive sampling was twofold. First, as described in the manuscript, purposive sampling supported consideration of diversity and representation of minorities within our interview sample. Second, we also selected this methodology as part of our strategy to select information-rich cases to illuminate a range of experiences among individuals with BD who self-reported psilocybin use (Patton, 2014).
- Understanding risk and safety factors for this understudied population were among the central aims to this broader explanatory sequential design study (Ivankova & Creswell, 2006; Creswell & Clark, 2018). Purposive sampling (Patton, 2014) provided a methodology to support these aims. Numerous team

meetings were held to discuss participant selection factors for phase II including willingness to participate in follow up, demographics, in addition to emphasizing recruitment of individuals who reported negative experiences, in order to balance representation.

- References:
- Ivankova NV, Creswell JW, Stick SL. Using mixed-methods sequential explanatory design: From theory to practice. Field methods. 2006;18(1):3-20.
- Creswell JW, Clark VLP. Designing and conducting mixed methods research. Third ed: Sage publications; 2018.
- Patton MQ. Qualitative research & evaluation methods: Integrating theory and practice: Sage publications; 2014.

***I couldn't find info concerning medications. Were patients under mood stabilizers, antidepressants, antipsychotics? Did they inform their current use of medication? If affirmative, what are the potential effects of psychedelics interacting with these medications? Some of them are likely to reduce the intensity of the effects, for instance.***

- Response: Thank you for your questions regarding medications. Given that the scope of Phase II was to describe subjective experiences of individuals living with BD who self-reported psilocybin use, and considering the discrete sample size common to qualitative follow up interview studies (Braun & Clark, 2021), preliminary reporting on medication use was limited to Phase I data collection and analysis. Morton and colleagues (2022) surveyed about psilocybin use in general, and not specific experiences. This limited their ability to analyze the contextual factors (e.g., including or discontinuation of particular psychiatric medications) surrounding each experience of adverse events. While they found 43.4% of survey respondents indicated that they were not taking any psychiatric medications at the time of psilocybin use, these results should be interpreted cautiously as self-report data from naturalistic settings limits specificity of potential time frame of drug interaction. We believe more research in this area is warranted, especially as you have highlighted, the possible reduction in effect. However, this was beyond the scope of the qualitative study described in this manuscript.
    - References:
    - Braun V, Clarke V. To saturate or not to saturate? Questioning data saturation as a useful concept for thematic analysis and sample-size rationales. Qualitative research in sport, exercise and health. 2021 Mar 4;13(2):201-16.
    - Morton E, Sakai K, Ashtari A, Pleet MM, Michalak EE, Woolley J. Risks and benefits of psilocybin use in people with bipolar disorder: An international web-based survey on experiences of 'magic mushroom' consumption. (In review). 2022.

***Authors wrote that "adults with BD underwent a range of experiences that could generally be categorized as either improving their mental health and functioning or eliciting undesired mental health impacts". This appears to be circular, since the subgroup was made of subjects who had very good and very bad experiences with the drug.***

- Response: Thank you for your comments and feedback. We agree this is a concern and have added the following caveat to highlight this issue: "We found that during and following full-dose psilocybin use, adults with BD underwent a range of experiences that could generally be categorized as either improving their mental health and functioning or eliciting undesired mental health impacts *(although it is important to note that our sampling procedure enriched for individuals who reported positive or negative outcomes after psilocybin use)*." (See lines 606-607.)

In conclusion, Dr. Ribeiro, it is our hope that these responses and corresponding manuscript modifications demonstrate our alignment with the publishing policies and quality that PLOS ONE stands for. Please do not hesitate to contact us with further clarification that may be needed by you or anyone on your team.

Take care.

Sincerely,

Meghan DellaCrosse
mdellacrosse@wi.edu | 646-853-1760
Doctoral candidate, The Wright Institute | Berkeley, CA
SFVA | UCSF affiliate | Translational Psychedelic Research Program (TrPR)
Ohio State University | Wexner Medical Center Early Psychosis Research | EPICENTER
